# NFAT5 genes are part of the osmotic regulatory system in Atlantic salmon (*Salmo salar*)

**DOI:** 10.1016/j.margen.2016.06.004

**Published:** 2017-02

**Authors:** Marlene Lorgen, Even H. Jorgensen, William C. Jordan, Samuel A.M. Martin, David G. Hazlerigg

**Affiliations:** a Institute of Biological and Environmental Sciences, University of Aberdeen, AB24 2TZ, UK; b Department of Arctic and Marine Biology, Faculty of BioSciences Fisheries & Economy, University of Tromsø, Norway; c Zoological Society London, Institute of Zoology, London NW1 4RY, UK

**Keywords:** Nuclear activated factor of T-cells, Salinity, Salmonid, Thyroid hormone, Whole genome duplication

## Abstract

The anadromous Atlantic salmon utilizes both fresh and salt water (FW and SW) habitats during its life cycle. The parr-smolt transformation (PST) is an important developmental transition from a FW adapted juvenile parr to a SW adapted smolt. Physiological changes in osmoregulatory tissues, particularly the gill, are key in maintaining effective ion regulation during PST. Changes are initiated prior to SW exposure (preparative phase), and are completed when smolts enter the sea (activational phase) where osmotic stress may directly stimulate changes in gene expression. In this paper we identify 4 nuclear factor of activated T cells (NFAT5, an osmotic stress transcription factor) paralogues in Atlantic salmon, which showed strong homology in characterized functional domains with those identified in other vertebrates. Two of the identified paralogues (NFAT5b1 and NFAT5b2) showed increased expression following transfer from FW to SW. This effect was largest in parr that were maintained under short day photoperiod, and showed the highest increases in chloride ion levels in response to SW exposure. The results of this study suggest that NFAT5 is involved in the osmotic stress response of Atlantic salmon.

## 1 Introduction

The Atlantic salmon is an anadromous species, spending the first one to three years of life in a freshwater (FW) environment before migrating downstream and out to sea for one or multiple winters before returning to its natal stream to spawn. A process termed the parr-smolt transformation (PST) facilitates this exploitation of FW and salt water (SW) environments. During PST, FW juveniles called parr undergo numerous physiological changes to become saltwater adapted ‘smolts’ (McCormick, 2013). The success of PST is vital to survival during FW to SW transfer, which occurs in a synchronized fashion in response to increasing photoperiod, with the aim of entering the sea during the narrow ‘smolt window’ in the spring when SW survival rates are highest.

The pathways governing detection of environmental salinity via molecular osmotic sensors in teleosts is not clear, however, a number of candidate genes that may serve this function have been identified, including adenyl cyclase (Saran and Schaap, 2004) and calcium sensing receptor (CaSR) (Nearing et al., 2002). The expression of downstream target genes is assumed to be modulated by osmotically-regulated transcription factors (Fiol and Kültz, 2007). One such transcription factor is the osmotic response element binding protein (OREBP), also known as tonicity response element binding protein (TonEBP) or nuclear factor of activated T-cells 5 (NFAT5). NFAT5 is the most ancestral of the NFAT gene family, showing high similarity to the single NFAT genes identified in *Drosophila melanogaster* and in pearl oyster (Graef et al., 2001, Huang et al., 2015), with high homology in the DNA-binding domain and the rel-homology domain (RHD). The NFAT5 DNA binding domain within the RHD regulates osmotic responses by binding to osmotic response elements (OREs) (Cheung and Ko, 2013).

During osmotic stress, changes in extracellular tonicity cause rapid changes in nuclear abundance of NFAT5 via nucleocytoplasmic trafficking mechanisms, with hyper-tonicity inducing nuclear accumulation of NFAT5 and hypo-tonicity resulting in nuclear export in mammals (Ko et al., 2000, Woo et al., 2000). In addition, increased NFAT5 mRNA levels have been observed during hyper-osmotic stress in mammals (Ko et al., 2000). Although hyper-tonically induced nuclear transport acts to generate a prompt response in downstream gene transcription, increased NFAT5 synthesis is also important for sustaining osmo-adaptation in the presence of chronic hyper-tonic stress (Cheung and Ko, 2013).

NFAT5 is involved in osmo-sensory signal transduction in killifish (*Fundulus heteroclitus*) gill, binding to OREs in the promoter of iodothyronine deiodinase 2 and initiating transcription in response to hypo-osmotic stress (Lopez-Bójórquez et al., 2007). Many of the physiological changes occurring during PST in the Atlantic salmon are directly or indirectly regulated by thyroid hormones (THs) (Dickhoff et al., 1978, Hoar, 1988, Lorgen et al., 2015), which require conversion from pro-hormone thyroxine (T4) to active thyroid hormone triiodothyronine (T3) to become functional (Darras and Herck, 2012). The iodothyronine deiodinase (dio) gene family acts to locally regulate the availability of T3 and the action of dio2 results in increased T3 availability in vertebrates (Darras and Herck, 2012).

To date, no NFAT5 genes have been characterized in the salmonid family. In this paper we have identified a repertoire of 4 NFAT5 paralogues in the Atlantic salmon, which show high similarity in structure to those characterized in other vertebrates. We show an increase in mRNA expression of two of the paralogues following 24 hour SW challenge in vivo and hypothesize that these genes may act to mitigate osmotic stress during FW to SW transition in salmonid smolts.

## 2 Methods

### 2.1 Characterisation of NFAT5 repertoire in Atlantic salmon

#### 2.1.1 NFAT5 paralogue identification in the Atlantic salmon genome

Searches for teleost NFAT5 nucleotide sequences on NCBI and Ensembl revealed the presence of 2 NFAT5 paralogues in teleosts; NFAT5a and 5b. Blast searches were carried out against the Atlantic salmon genome (*Salmo salar* Linnaeus, 1758; Taxid: 8030, version AGKD00000000.4, Lien et al., 2016) on NCBI using BlastN default parameters and available NFAT5a and NFAT5b nucleotide sequences from *Takifugu rubripes* (ENSTRUG00000011018.1 and ENSTRUT00000045105, respectively) to identify homologous genes in *S*. *salar*. BlastN was also used to search the Rainbow Trout (*Oncorhynchus mykiss*) (CCAF00000000.1, Berthelot et al., 2014) and Northern Pike (*Esox lucius*) (AZJR00000000.2, Rondeau et al., 2014) WGS databases for NFAT5 paralogues using the full length Atlantic salmon sequences obtained from searching the *S*. *salar* WGS database.

For each paralogue identified, the intron/exon structure was determined using GENSCAN (http://genes.mit.edu/GENSCAN.html) (Burge and Karlin, 1997) combined with manual alignment and the amino acid sequences were generated by translation with ExPASY (Gasteiger et al., 2003). Multiple sequence alignment of predicted amino acid sequences was performed using CLUSTALW2 (http://align.genome.jp) (Larkin et al., 2007).

Synteny analyses were carried out using the Generic Genome Browser (version 2.55) on SalmoBase (http://salmobase.org/cgi-bin/gb2/gbrowse/salmon_GBrowse_Chr_NCBI/). The data source for the browser was Ssal ICSASG_v2. 100 kbp up- and downstream of each *S*. *salar* NFAT5 paralogue were analysed along with the same region in *Esox Lucius* (NCBI Genome Data Viewer, data source ASM72191v2), *Takifugu rubripes*, *Lepisosteus oculatus*, *Xenopus* and *Mus musculus* (Ensembl genome browser) NFAT5a and NFAT5b.

#### 2.1.2 Identification of conserved NFAT5 protein domains

Conserved domains (as described in Cheung and Ko, 2013) were identified by amino acid alignments with *Homo sapiens* NFAT5 protein isoform c. NFAT5 consists of a rel-like homology domain (RHD), a canonical nuclear export signal (NES), an auxiliary export domain (AED), a dimerization domain (DD) within the RHD, and 3 transactivation domains (AD1, AD2, AD3), AD1 at the N-terminal and AD2 and AD3 at the C-terminal (Tong et al., 2006, Lopez-Ródriquez et al., 2001). Phylogenetic trees predicting evolutionary relationships were generated with MEGA6 software (Tamura et al., 2013) using the amino acid sequence alignment of the RHDs with the neighbour-joining method and 10,000 × iteration of bootstrapping (Fig. S1B).

### 2.2 SW challenge experiments

Fertilized Atlantic salmon eggs from a commercial hatchery (Aquagen, Kyrksӕterøra, Norway) were raised at the University of Tromsø Aquaculture research station. Fish were held at 10 °C under constant light (LL) from the free feeding stage and fed continuously with pelleted salmon food (Skretting, Stavanger, Norway) using automatic feeders. Photoperiod manipulation was carried out to generate fish that were either prime smolt condition and able to osmoregulate well, or those that were maintained under photoperiod conditions that meant they were poor at osmoregulating. At the start of the experiment, fish were either maintained under LL or transferred to short-day photoperiod (SP, 8L:16D). After 8 weeks under SP a subset of fish from the SP group were switched back to LL (to stimulate PST). Fish were transferred to SW for 24-hr (n = 6) at the time points indicated in before sampling, and FW individuals were also sampled (n = 6) as time matched controls. Euthanization was by overdose with 0.05% v/v aqueous 2-phenoxyethanol (Sigma Aldrich, UK). Gills were collected in RNA-Later for subsequent RNA extraction. Blood was taken from the caudal vein into heparinised tubes and centrifuged at 500 ×* g* for 15 min to collect plasma, and sodium, potassium and chloride were analysed with ion selective electrodes using standard solution on the COBAS c111 auto analyser (Roche Diagnostics, Norway). Length and weight measurements were also taken throughout the duration of the study as an indication of successful PST by way of a reduction in condition factor which was calculated using the equation, CF = body weight in grams × 100 × fork length (in cm)^^− 3^.

### 2.3 NFAT5 gene expression analysis by qPCR

#### 2.3.1 RNA extraction and cDNA synthesis

Total RNA was extracted from 50 mg of gill tissue, which was homogenized in TRI-reagent (Invitrogen) using tungsten carbide beads (3 mm, Qiagen) in a mixer mill MM30 (Retsch) following the manufacturer's instructions. The resulting RNA pellet was washed twice with cold 80% ethanol and dissolved in nuclease-free water (Sigma). RNA concentration was determined by a nanodrop ND1000 spectrophotometer (LabTech) and RNA integrity by the Agilent Bioanalyser 2100. RNA was stored at − 80 °C until required for cDNA synthesis.

Synthesis of cDNA was performed using the Quantitect cDNA Synthesis kit (Qiagen) starting with 2 μg of total RNA, according to the manufacturer's protocols, briefly described here. 4 μl of gDNA wipe-out was added to 2 μg total RNA in a total volume of 24 μl with water and incubated at 42 °C for 2 min to remove any genomic DNA contamination. A mastermix consisting of 8 μl buffer, 2 μl primer and 2 μl reverse transcriptase per reaction was then added to the treated RNA and incubation at 42 °C continued for another 25 min before a final 5 minute incubation at 95 °C. cDNA was diluted to a final volume of 100 μl representing an original concentration of 500 ng μl^− 1^ RNA before use in subsequent PCR and qPCR assays.

#### 2.3.2 Measurement of RNA expression by qPCR assay

The mRNA expression of 4 NFAT5 paralogues was assayed by real time PCR. Confirmation of primer (Table S1) specificity was by sequence analysis. A volume of 3 μl of cDNA was used as template in a final volume of 20 μl with 10 μl of 2 × GoTaq® SYBR-green qPCR master mix (Promega), 5 μl nuclease-free water (Promega) and 2 μl of primers. qPCR was carried out in 96 well plates on a DNA Engine Opticon™ 107 System (MJ Research Inc.). The PCR cycles were 95 °C for 5 min then 40 cycles of 95 °C for 30 s, 60–65 °C for 30 s and 72 °C for 30 s, with a final extension at 72 °C for 7 min. Primer specificity was further confirmed by the presence of a single peak in a melting curve with reads every 0.5 °C from 70 and 92 °C.

RNA expression was calculated from a standard curve generated by plotting log dilution against threshold cycle number (C(t)) obtained from a dilution series ran in the same plate as the plate of interest. Efficiency was calculated as E = 10^^(− 1/slope)^ using serial dilutions, where slope was obtained from a plot of C(t) against log input cDNA concentration. Expression levels were normalized against 2 reference genes: elongation factor 1α (Elf-1α) and beta actin (β-actin).

#### 2.3.3 Statistical analysis

To determine if NFAT5 mRNA expression or plasma chloride levels were significantly modulated in response to 24 hour SW challenge over time, statistical analysis was carried out by way of 2-way ANOVA where factors were treatment and sampling date. The models were tested using diagnostic plots of the residuals in R. Post-hoc testing in the form of Tukey's multiple comparison test was carried out where appropriate against relative mRNA expression in FW controls. p Values < 0.05 were considered to be significant.

## 3 Results

### 3.1 Four NFAT5 paralogues identified in Atlantic salmon

Four NFAT5 paralogous genes named NFAT5a1, NFAT5a2, NFAT5b1 and NFAT5b2 were identified in the Atlantic salmon genome version AGKD00000000.4: NFAT5a1 on chromosome ssa10 (AGKD04000113.1, 88550193..88575551, E = 4e^− 136^), NFAT5a2 on chromosome ssa16 (AGKD04000076.1, 22037579..22059470, E = 5e^− 122^), NFAT5b1 on chromosome ssa11 (AGKD04000127.1, 18800113..18865778, E = 6e^− 108^) and NFAT5b2 on chromosome ssa26 (AGKD04000059.1, 19149934..19204506, E = 2e^− 94^). BlastN searches of the *O*. *mykiss* genome also revealed four NFAT5 paralogues (NFAT5a1, NFAT5a2, NFAT5b1 and NFAT5b2). Only two NFAT5 paralogues were identified in the *E*. *lucius* genome (NFAT5a and NFAT5b).

**Fig. 1:**
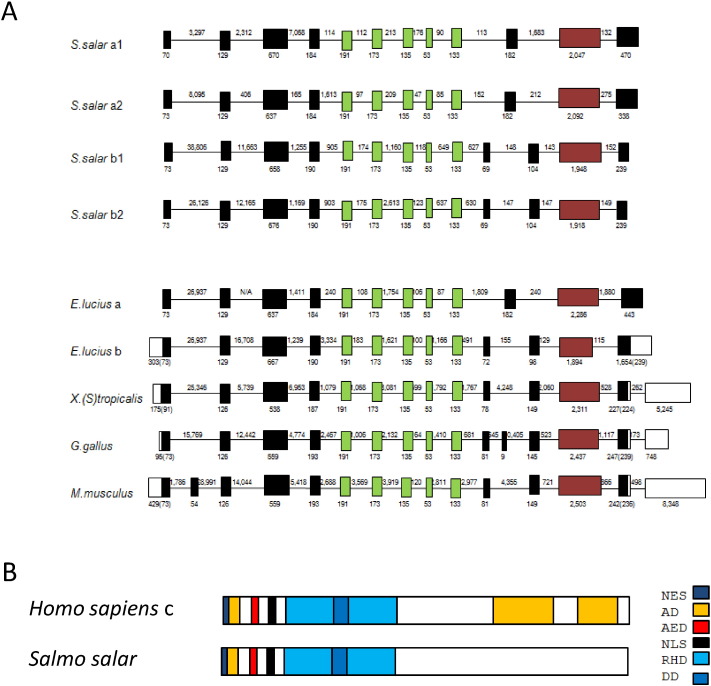
*S*. *salar* NFAT5 paralogue structure. (A) Intron/exon structure of *S*. *salar* NFAT5 paralogues and vertebrate orthologues. Intron/exon structure was determined using GENSCAN. Open boxes indicate non-coding exons, filled boxes are coding exons and connecting lines represent introns. Green boxes indicate 100% conserved exon sizes across vertebrates, black are less conserved and red indicates the highly variable 3′ region. Numbers above lines are intron sizes while those below are exon sizes with the coding sequence in brackets for those exons which are not completely coding. Incomplete intron sizes where introns cross multiple contigs. (B) Schematic diagram of conserved domains in NFAT5 in order from 5′ to 3′ are NES (AD1), AED, NLS, RHD (DD), AD2 and AD3 (from Cheung and Ko, 2013). Conserved domains were identified by alignment of *S*. *salar* NFAT5 amino acid sequences with *H*. *sapiens* NFAT5 isoform c (NP_006590.1).

Intron-exon structure was determined in the CDS of *S*. *salar* paralogues and in mRNA sequences of other vertebrates (Fig. 1A). All splice donor/acceptor sites for introns followed the consensus ‘GT/AG’ rule. There is a highly conserved core of 5 exons (green boxes) which is 100% conserved in length and a highly variable 3′ region where little alignment is observed (red). 5′ exons are highly conserved in length with a few variations, as is the case with the exons between the core and variable regions (black).

### 3.2 NFAT5 protein structure

Vertebrate NFAT5 proteins have a number of conserved regions described in the Methods section. Alignments of *S*. *salar* NFAT5s with *H*. *sapiens* NFAT5 protein isoform c revealed conservation of the NES, AD1, AED, NLS, RHD and DD domains, but no alignment was observed for AD2 and AD3 (Fig. 1B). Clustal alignments of conserved domains are presented in Fig. S1. The highly conserved “core” of 5 exons in green (Fig. 1A), contains the key RHD domain. 78% of all amino acids in the RHD were conserved between *H*. *sapiens* and all *S*. *salar* paralogues. The C terminal of the protein is more variable in length and in sequence, and contains regions rich in glutamine amino acids.

### 3.3 NFAT5 phylogeny and NFAT5 duplications

**Fig. 2:**
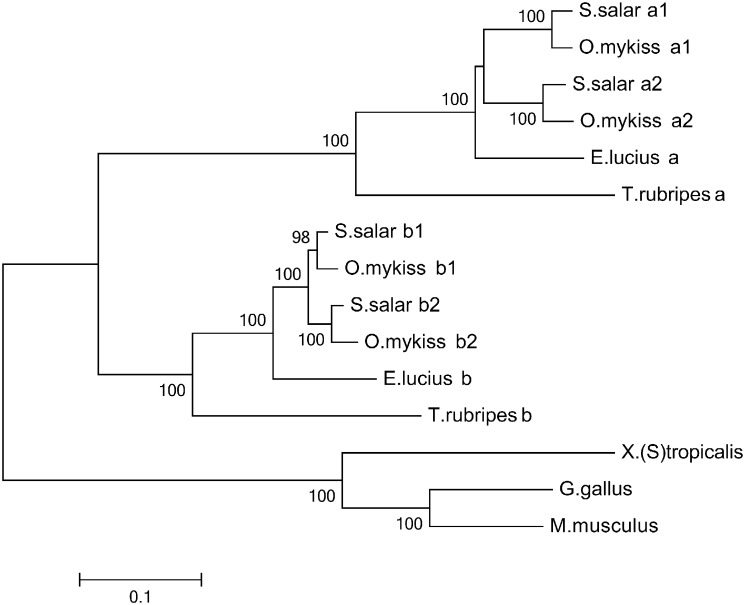
Vertebrate NFAT5 phylogenetic tree. Phylogenetic tree showing the evolutionary relationship between NFAT5 genes across vertebrates. Nucleotide alignment was generated in ClustalW2 in MEGA6 using only the 5 core exons comprising the RHD. Accession numbers for nucleotide sequences used to generate the tree were as follows; *M*. *musculus* (NM_018823.2), *Gallus gallus* (NM_001199000.1), *X*. *tropicalis* (XM_004913614.1), *T*. *rubripes* a (XM_003967127.1), *E*. *lucius* a (AZJR02001609.1), *S*. *salar* a1 (AGKD04000113.1), *S*. *salar* a2 (AGKD04000076.1), *T*. *rubripes* b (ENSTRUT00000045105), *E*. *lucius* b (AZJR02001577.1), *S*. *salar* b1 (AGKD04000127.1) and *S*. *salar* b2 (AGKD04000059.1). The tree was constructed using the neighbour joining method in MEGA 6 and bootstrapped 10,000 times; only values over 75% are shown.

A phylogenetic analysis was carried out using the amino acid sequence alignment of the RHDs using the neighbour-joining method and 10,000 × iteration of bootstrapping and the results are shown in Fig. 2. Teleost NFAT5a and NFAT5b form two distinct clusters, separate from the non-teleost single NFAT5 gene. Within the NFAT5a and NFAT5b clusters, the salmonid NFAT5a1/a2 and NFAT5b1/b2 paralogues cluster together with the single *E*. *lucius* NFAT5a and NFAT5b genes as the most common ancestor in each case. Synteny analysis (Fig. S2) revealed a number of syntenic genes between NFAT5a loci and between NFAT5b loci (white).

Both *S*. *salar* NFAT5a paralogues shared synteny with *L*. *oculatus*, *T*. *rubripes* and *E*. *lucius* NFAT5a loci and the same was true for the NFAT5b paralogues. mical2a, dkk3a, usp47, clec3a and vat11 (shown in *T*. *rubripes* NFAT5b) were present further upstream of *S*. *salar* NFAT5b paralogues beyond the 100 kbp cut-off. cyb5 (shown in *Xenopus*) was also identified in *M*. *musculus* and *L*. *oculatus* outside the 100 kbp cut-off, as was psmd7 in Mouse. Furthermore, cy5b could also be identified in Fugu NFAT5b and in *S*. *salar* NFAT5bs, cyb5a was identified outside the cut-off. Reduced synteny was observed between NFAT5a and b loci (blue), and overall a higher degree of synteny was observed between NFAT5b and NFAT5 in other vertebrate species.

### 3.4 Differential tissue distribution of expression of NFAT5 paralogues

**Fig. 3:**
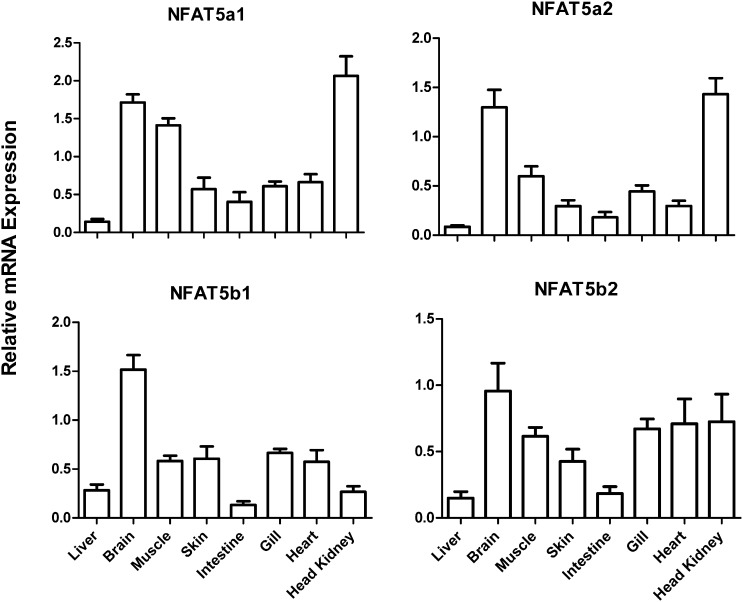
Tissue distribution of NFAT5 paralogue mRNA in *Salmo salar*. mRNA expression was determined by qPCR and normalized to reference gene expression. Columns show mean normalized expression and error bars represent the standard error of the mean (SEM, n = 5).

Distinct differences were observed in tissue distribution of expression between the NFAT5a and the NFAT5b paralogues in freshwater acclimated fish (Fig. 3). NFAT5a1 showed highest expression levels in brain, muscle and head kidney, NFAT5a2 in brain and head kidney, while NFAT5b1 showed highest expression in the brain and NFAT5b2 showed a more uniform distribution of expression between tissues.

### 3.5 Modulation of NFAT5 expression following 24 hr SW challenge

**Fig. 4:**
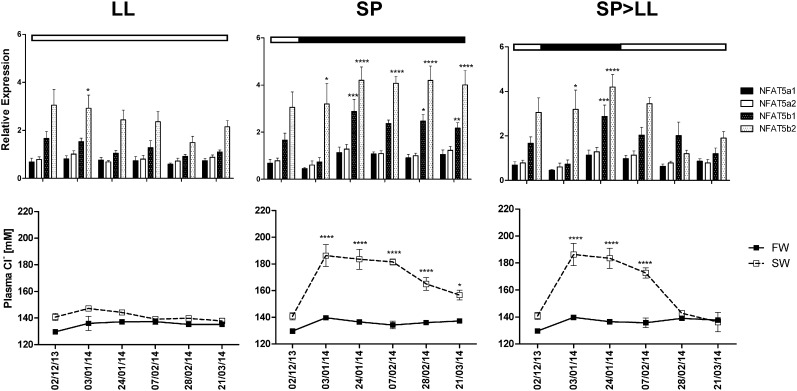
NFAT5 mRNA expression in 24-hr SW challenged fish. mRNA expression was determined by qPCR and normalized to reference gene expression. Top row: NFAT5 mRNA data are presented as normalized SW expression levels relative to normalized FW control levels under each photoperiod regime. Bottom row: data presented are a measure of osmotic stress, plasma chloride levels (in mM) under each photoperiod regime. The bars at the top of each graph represent photoperiod regime (open bars = LL, filled bars = SP). All error bars show SEM (n = 6).

In the SW challenge experiment, individuals were transferred to SW for 24 h before sampling following exposure to different photoperiods for various lengths of time (Fig. 4, see materials for full experimental design). Fish maintained in FW were also sampled after 24 h to act as controls. Condition factor was determined for each fish sampled throughout the study and was significantly affected by date (F_(5,198)_ = 3.423, p < 0.01) and the interaction between date and photoperiod regime (F_(10,__198)_ = 3.159, p < 0.001) with a highly significant decline observed following transfer from SP to LL from 1.371 ± 0.037 to 1.12 ± 0.016 (p < 0.0001). Plasma chloride ion levels showed a significant response to an interaction of treatment and sampling date (F_(25,180)_ = 6.620, p < 0.0001).

NFAT5a1 and a2 showed no significant response to SW challenge under any photoperiod condition, however, a gradual and significant increase in NFAT5a2 expression was observed throughout the duration of the study, irrespective of salinity (F_(5,171)_, p < 0.0001), resulting in a significant increase in expression between the initial and final sampling points under all conditions with the exception of the fish that remained under SP in FW (Fig. S3).

A significant interaction between treatment and sampling date was identified for NFAT5b1 expression (F_(25,170)_ = 2.259, p = 0.012), which reached a maximum 3 fold increase when held under SP during SW challenge (p < 0.0001) and also significantly increased in response to SW challenge when held under SP > LL (p < 0.01).

A significant interaction of treatment and sampling date was also observed in NFAT5b2 expression (F_(25,173)_ = 1.633, p = 0.0366). NFAT5b2 expression increased in response to SW challenge under all photoperiod conditions (p < 0.0001), with the most distinct up-regulation observed in fish held under SP, with a 4 fold change observed compared to FW controls Under LL, NFAT5b2 fold change steadily declined throughout the study which under SP they remained elevated. In the SP > LL group, fold change between FW and SW NFAT5b2 declined sharply following transfer to LL.

## 4 Discussion

### 4.1 Four NFAT5 paralogues retained following salmonid 4R WGD

The Salmonidae have undergone four rounds of genome duplication during their evolutionary history, and the results of phylogenetic and synteny analyses suggests that NFAT5a and NFAT5b arose following the third round (3R) whole genome duplication event which occurred in teleosts around 320–350 mya (Christoffels et al., 2004), while the duplicated versions of the a and b paralogues likely arose from a relatively recent fourth round (4R) whole genome duplication event which occurred specifically in salmonids around 88 to 103 mya (MacQueen and Johnston, 2014). Atlantic salmon are categorized as pseudo-tetraploid and are in the process of diploidization to return to diploid form (Davidson et al., 2010, Lien et al., 2016, Berthelot et al., 2014). During diploidization, duplicated paralogues are often lost, silenced, or can become sub- or neo-functionalized (Mungpakadee et al., 2008). In the case of *S*. *salar* NFAT5, all 4 paralogues appear to be expressed, however, only the NFAT5b paralogues showed differential expression in response to SW challenge, which suggests that some degree of sub-functionalization may have occurred in the regulation of expression of NFAT5 paralogues.

### 4.2 Conserved vertebrate NFAT5 domains present in Atlantic salmon

The NFAT gene family are characterized by a highly conserved RHD, which we have identified in 4 NFAT5 paralogues in *S*. *salar*. Unlike NFATs 1–4, NFAT5 proteins lack a calcineurin binding regulatory domain, but have long C terminals, which in mammals contain 2 conserved transactivation domains, which we found to be absent, or non-conserved in *S*. *salar* despite all paralogues having a long C terminal. The RHD region is responsible for DNA binding of the ORE/TonE motif in target promoters, while the ADs have been implicated in promoting transcription (Cheung and Ko, 2013). The lack of conserved C terminal ADs in *S*. *salar* infers reduced transcriptional activity, or evolution of species/class specific ADs. Tong et al. (2006) characterized 3 protein domains involved in nucleocytoplasmic shuttling in response to changes in extracellular tonicity; NED, AED and NLS. The NLS plays an important role in nuclear import, while the NES is primarily responsible for nuclear export, specifically under isotonic conditions and the AED is important for hypo-tonicity induced nuclear export.

### 4.3 NFAT5 plays a role in the osmotic stress response in Atlantic salmon

Rapid salinity transfer causes osmotic stress in teleost fish which results in changes in cellular volume and ionic concentration (Evans et al., 2005, McCormick, 2013). Failure to osmoregulate (determined by increased plasma chloride ion concentration) following 24 h transfer to SW was found to be enhanced in Atlantic salmon held under constant short day photoperiod. These fish showed no decease in condition factor across the sampling schedule, suggesting a failure to smolt. The increase in plasma chloride levels in these ‘poor smolts’ was accompanied by increased NFAT5b1 and NFAT5b2 mRNAs, suggesting that increased transcription of NFAT5bs may have a role in mitigating osmotic stress in Atlantic salmon.

In contrast to fish maintained under short day photoperiod until the end of the study, those transferred to long photoperiod were not osmotically stress and showed declining NFAT5b response. In mammalian cells, extracellular osmotic stress stimulates adaptive cellular responses such as accumulation of organic osmolytes to maintain stasis in intracellular electrolyte levels (Burg et al., 1997), induction of heat shock protein 70 (HSP-70) expression which protects cells from stress-induced apoptosis (Shim et al., 2002), and regulation of developmental processes, such as neuron development via stimulation of inositol (Maouyo et al., 2002).

The genes bringing about these adaptations to hyper-tonicity are transcriptionally regulated by monomeric OREs in target gene promoters (Lopez-Ródriquez et al., 2001), of which multiple copies in close proximity may be required to be fully functional (Ko et al., 1997). A variable increase in expression of the recently characterized Atlantic salmon dio2a paralogue in gill primary lamellae in response to hyperosmotic challenge following PST has been observed, and investigation of the dio2a promoter uncovered enrichment for OREs (Lorgen et al., 2015), suggesting that dio2a may be an NFAT5 regulated osmotic response gene in Atlantic salmon.

A study by Lopez-Bójórquez et al. (2007) in the euryhaline killifish observed increased dio2 activity and nuclear recruitment of a putative ORE-BP (NFAT5) in response to hypo-tonic stress in the liver in SW-adapted individuals. Furthermore, in FW-acclimated rainbow trout, hyper-osmotic stress induced a decrease in dio2 expression in the liver (Orozco et al., 2002). These results suggest that NFAT5 trafficking and expression could be species and/or tissue specific in teleosts. Brennan et al. (2015) showed that transcriptomic responses to osmotic stress in killifish were influenced by natural habitat salinity, and that the same genes could act in different ways dependent upon the natural salinity of the habitat of the individual.

Although we did observe transcriptional regulation of NFAT5b paralogues following 24 hour SW exposure, rapid and transient changes in expression in the first minutes and hours after transfer to SW may have been missed. Fiol and Kültz (2005) identified two transcription factors, osmotic stress transcription factor I (OSTF1) and the tilapia homolog of transcription factor II B (TFIIB), which showed rapid and transient induction during hyperosmotic stress, reaching maximum expression levels only 2 h after SW transfer. Future studies utilising a more intensive sampling scheme covering the immediate and early period post transfer may uncover further regulation of NFAT5 paralogues.

### 4.4 Conclusion

Four NFAT5 paralogues were identified in Atlantic salmon, which share strong homology with other vertebrates in characterized functional domains. Differential distribution of expression between tissues and in response to 24 hour SW was observed between paralogues. An sustained increase in NFAT5b1 and NFAT5b2 mRNA abundance in response to SW challenge in fish held under SP, a group of fish that were unable to osmoregulate efficiently and displayed pronounced osmotic stress, suggesting that increase NFAT5b mRNA expression plays a role in mitigating osmotic stress in Atlantic salmon. Previous work identified OREs in the dio2a promoter in Atlantic salmon which may bind NFAT5 in response to osmotic stress, inducing a downstream increase in the availability of local active thyroid hormone and subsequently switching on thyroid activated genes which promote SW adaptation in the Atlantic salmon gill.

The following are the supplementary data related to this article.

Fig. S1 Conserved NFAT5 protein domains. Clustal alignments of *S*. *salar* NFAT5 conserved domains identified with respect to *H*. *sapiens* NFAT5 protein isoform c (Cheung and Ko, 2013). Colours correspond to those in Fig. 1B. Fig. S1

Fig. S2 Synteny analysis of NFAT5 loci. 100 kbp up and downstream of *M*. *musculus*, *Xenopus*, *L*. *oculatus*, *T*. *rubripes*, *E*. *Lucius* and *S*. *salar* NFAT5 genes was analysed for syntenic genes (not to scale). Arrows indicate the direction of transcription with the following colour coding; blue: present in NFAT5a and NFAT5b, white: present in NFAT5a/NFAT5b only, black: non-syntenic gene, grey: hypothetical (predicted) gene. Fig. S2

Fig. S3 NFAT5a2 relative mRNA expression during 24 hour SW challenge experiment. mRNA expression was determined by qPCR and normalized to reference gene expression. Data are presented as normalized mRNA expression of individuals in FW controls and 24-hr SW transfer at each time point under each photoperiod regime. Expression modulation was independent of state of osmotic stress. All error bars show SEM (n = 6). Fig. S3

Table S1 *S*. *salar* NFAT5 and reference gene primer sequences and properties. Table S1
