# Supplementary material for: NFAT5 genes are part of the osmotic regulatory system in Atlantic salmon (Salmo salar)
Source: Mar Genomics. 2017 Feb;31:25–31. doi: 10.1016/j.margen.2016.06.004 (PMC5292104; doi:10.1016/j.margen.2016.06.004)
Supplement: Fig. S1 — Conserved NFAT5 protein domains. Clustal alignments of S. salar NFAT5 conserved domains identified with respect to H. sapiens NFAT5 protein isoform c (Cheung and Ko, 2013). Colours correspond to those in Fig. 1B. [file mmc1.pdf]

FIG. S1

```
S.salar_a1      MPSDFISL-SSDLDLNSPKSLYSKESVYDLLPKELQLQSSSSQTDTPTMSQKSGGEAGPPPSASLASDATSSTSSPS
S.salar_a2      MPSDFISLLSADLDLNSPKSLYSKESVYDLLPKELQLQSSSSQTDTPTMSQKSGGEAGLPPSAAMASATSSSPCPSS
S.salar_b1      MPSDFISLLSADLDLNSPKSLYSKESVYDLLPKELQLPSSTQQNPVAAMSQKSGGEAVPPPSAALDSDATTVSSSMT
S.salar_b2      MPSDFISLLSADLDLNSPKSLYSKESVYDLLPKELQLPSSTQQNPVAAMSQKSGGEAVPPPSAALDSDATTVSSSMT
H.sapiens_c     MPSDFISLLSADLDLESPKSLYSRESVYDLLPKELQLPPS-RETSVASMSQTSGGEAGSPPPAVVAADASSAPSSSS
                ******** * **** ******* *************  *        ***.*****  **.* : : ::: ... :

S.salar_a1      QNTPSKRRPVLSISPPPEDLFDDSS          S.salar_a1      RKSRKRTPRQRPGPKPAP
S.salar_a2      QNTPSKRRPVLSISPPPEDLFDDSS          S.salar_a2      RKSRKRTPRQRPGAKPAP
S.salar_b1      QMTPSKRRTVLNISPPPEDLFDDSR          S.salar_b1      RKSRKRTPRQRPGPKPAS
S.salar_b2      QMTPSKRRTVLNISPPPEDLFDDSR          S.salar_b2      RKSRKRTPRQRPGPKPAS
H.sapiens_c     HPSTPKRHTVLYISPPPEDLLDNSR          H.sapiens_c     RKSRKRNPKQRPGVKRRD
                : :..**:.** ********:*:*                           ******.*:**** *

S.salar_a1      LKGCLLSGQYPQKSEGKELKILLQPETQHRARYLTEGSRGSVKDRTQQGFPTVKLEGVSEPVVLQVFVASDTGRVKPHGFYQAC
S.salar_a2      LKGGLLSGQCPQKSEGKELKILVQPETQHRARYLTEGSRGSVKDRTQQGFPTVKLEGVSEPVMLQVFVASDTGRVKPHGFYQAC
S.salar_b1      QKGGTLASQFPQKSEGKELKILVQPETQHRARYLTEGSRGSVKDRTQQGFPTLKLEGVNEAVVLQVFVGNDAGRVKPHGFYQAC
S.salar_b2      QKGGTLASQFPQKSEGKELKILVQPETQHRARYLTEGSRGSVKDRTQQGFPTLKLEGVNEAVVLQVFVGNDAGRVKPHGFYQAC
H.sapiens_c     KKSPMLCGQYPVKSEGKELKIVVQPETQHRARYLTEGSRGSVKDRTQQGFPTVKLEGHNEPVVLQVFVGNDSGRVKPHGFYQAC
                 *.  *..* * *********::*****************************:**** .*.*:*****..*:************

S.salar_a1      RVTGRNTKACKEVDIEGTTVIEVPLEPSNAMSLAVDCVGILKLRNADVEARIGVAGSKKKSTRARLAFRVNIPHADGSVLTLQT
S.salar_a2      RVTGRNTTACKEVDIEGTTVIEVPLEPSSAMSLAVDCVGILKLRNADVEARIGVAGSKKKSTRARLAFRVNIPQPDGSVLTLQT
S.salar_b1      RVTGRNTTACKEVDIEGTTVIEVSLDPSNNMTLAVDCVGILKLRNADVEARIGVAGSKKKSTRARLVFRVNIPRPDGSVLTLQT
S.salar_b2      RVTGRNTTACKEVDIEGTTVIEVSLDPSNNMTLAVDCVGILKLRNADVEARIGVAGSKKKSTRARLMFRVNIPRPDGSVLTLQT
H.sapiens_c     RVTGRNTTPCKEVDIEGTTVIEVGLDPSNNMTLAVDCVGILKLRNADVEARIGIAGSKKKSTRARLVFRVNIMRKDGSTLTLQT
                *******..************** *:**. *:*********************:************ ***** : ***.*****

S.salar_a1      TSSPILCTQPAGVPEILKKSLHSCSVRGGEELFIIGKNFLKGTKVLFHENPADDNSWQAEAEIDMELFHQNHVIVKVPPYHNLS
S.salar_a2      TSSPILCTQPAGVPEILKKSLHSCSVRGGEELFIIGKNFLKGTKVIFQENPADDDSWQAEAEIDMELFHQNHVVVTVPPYHSLS
S.salar_b1      PSSPILCTQPAGVPEILKKSLHSCSARGDEEVFIIGKNFLKDTKVIFQENVSDEKSWKAEAEIDMELFHQNHLIVRVPPYQNLA
S.salar_b2      PSSPILCTQPAGVPEILKKSLHSCSVRGGEEVFIIGKNFLKDTKVIFQENVSDEKSWKAEAEIDMELFHQNHLIVKVPPYQNPA
H.sapiens_c     PSSPILCTQPAGVPEILKKSLHSCSVKGEEEVFLIGKNFLKGTKVIFQENVSDESWKSEAEIDMELFHQNHLIVKVPPYHDQHI
                .************************.:* **:*:*******.***:*:** :*: .**::**************::* ****:.

S.salar_a1      VSSPVSVGVYIMTNAGRSHDVQPFTYTP
S.salar_a2      VSSAVSVGVYITTNAGRSHDIQPFTYTP
S.salar_b1      IASAVCVGIYVVTNAGRSHDVQPFTYTP
S.salar_b2      IASAVCVGIYVVTNAGRSHDVQPFTYTP
H.sapiens_c     NTLPVSVGIYVVTNAGRSHDVQPFTYTP
                :: .*.**:*: ********:*******
```
