# Supplementary figures and images for: NFAT5 genes are part of the osmotic regulatory system in Atlantic salmon (Salmo salar)
Source: Mar Genomics. 2017 Feb;31:25–31. doi: 10.1016/j.margen.2016.06.004 (PMC5292104; doi:10.1016/j.margen.2016.06.004)

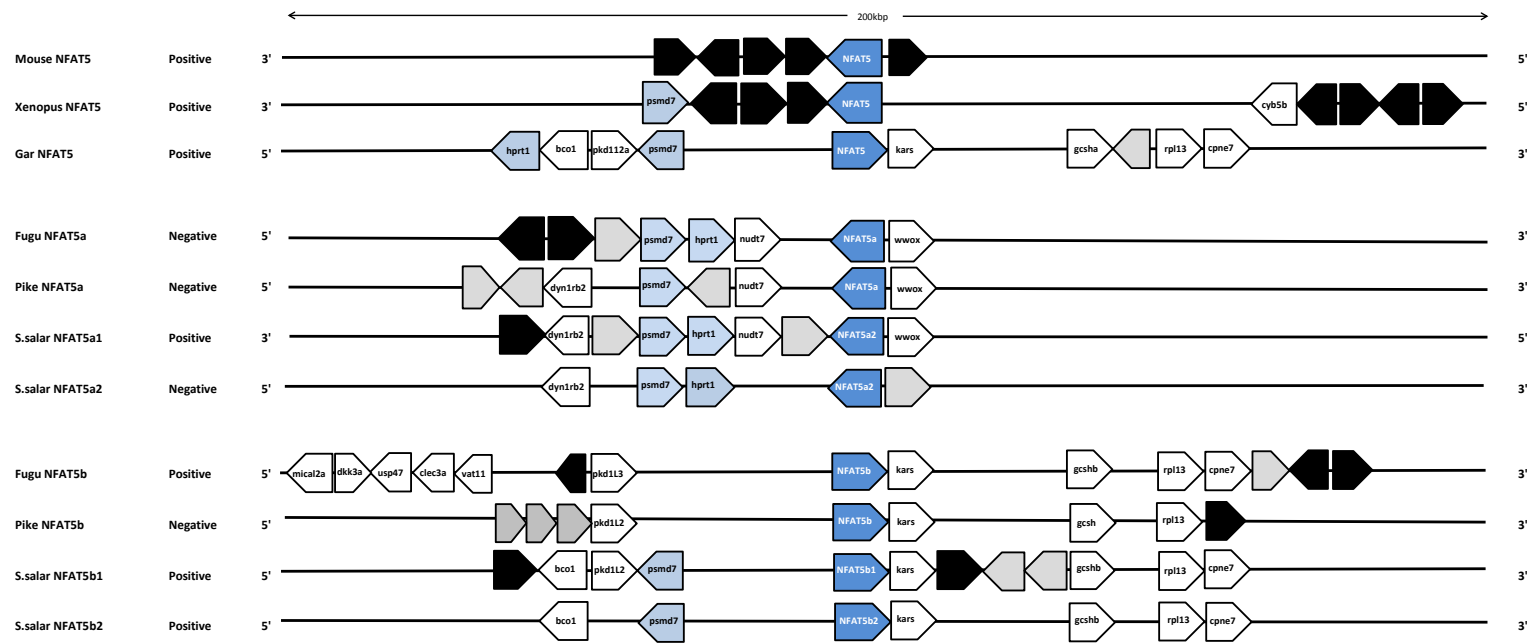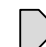

Supplement: Fig. S2 — Synteny analysis of NFAT5 loci. 100 kbp up and downstream of M. musculus, Xenopus, L. oculatus, T. rubripes, E. Lucius and S. salar NFAT5 genes was analysed for syntenic genes (not to scale). Arrows indicate the direction of transcription with the following colour coding; blue: present in NFAT5a and NFAT5b, white: present in NFAT5a/NFAT5b only, black: non-syntenic gene, grey: hypothetical (predicted) gene. [file mmc2.pdf]

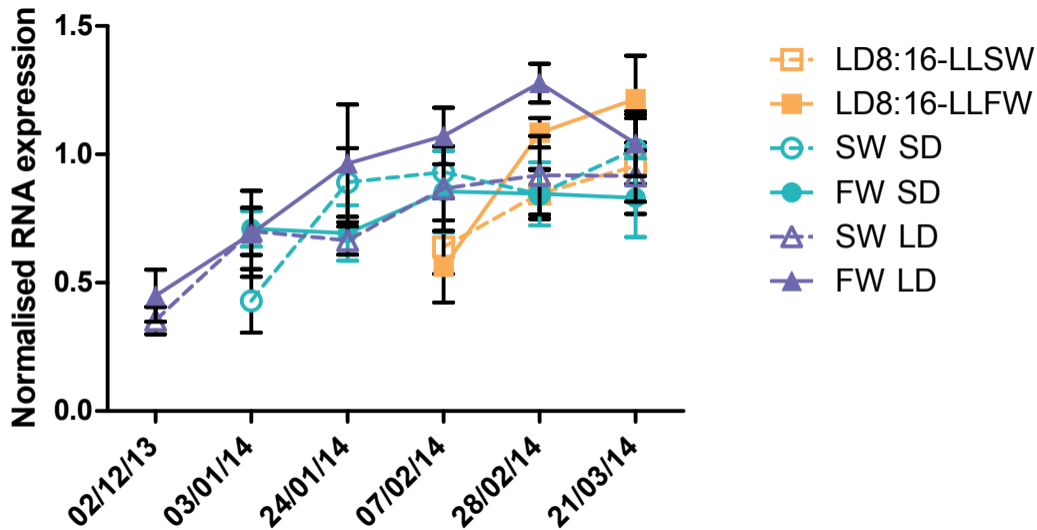

Supplement: Fig. S3 — NFAT5a2 relative mRNA expression during 24 hour SW challenge experiment. mRNA expression was determined by qPCR and normalized to reference gene expression. Data are presented as normalized mRNA expression of individuals in FW controls and 24-hr SW transfer at each time point under each photoperiod regime. Expression modulation was independent of state of osmotic stress. All error bars show SEM (n = 6). [file mmc3.pdf]
