# Supplementary material for: NFAT5 genes are part of the osmotic regulatory system in Atlantic salmon (Salmo salar)
Source: Mar Genomics. 2017 Feb;31:25–31. doi: 10.1016/j.margen.2016.06.004 (PMC5292104; doi:10.1016/j.margen.2016.06.004)
Supplement: Table S1 — S. salar NFAT5 and reference gene primer sequences and properties. [file mmc4.docx]

**Table S1**. *S. salar* NFAT5 and reference gene primer sequences and properties.

| Name | Sequence (5’ 🡪 3’) | Tm (^o^C) | Anneal (^o^C) | Size (bp) |
| --- | --- | --- | --- | --- |
| NFAT5a1_F | GTCGTCTGACACCCTCGTCTCG | 70.6 |  |  |
| NFAT5a1_R | GGAGGGGAAGACCGGGGACT | 71.7 | 65 | 139 |
| NFAT5a2_F | ACCCTCTGACGCCCTTATCTTA | 64.7 |  |  |
| NFAT5a2_R | GAGGGAAAGACAGGGGCAC | 66.0 | 62 | 138 |
| NFAT5b1_F | CAACAGCAACAGCAAATTCAG | 63.4 |  |  |
| NFAT5b1_R | GTTTCTGTTGTTGTTGTTGCTG | 62.2 | 60 | 259 |
| NFAT5b2_F | AGCAACTACAGCAACAGCAACT | 62.9 |  |  |
| NFAT5b2_R | GCTGCTGTTTCTGTTGTTGCCT | 67.6 | 60 | 315 |
| Elf-1α_F  Elf-1α_R  β-actin_F  β-actin _R | CAAGGATATCCGTCGTGGCA  CCTCTTGGTCGTTTCGCTGT  ATGGAAGATGAAATCGCCGC  TGCCAGATCTTCTCCATGTCG | 68.1  66.5  68.2  67.6 | 63  63 | 317  260 |
